# Acceleration for Efficient Automated Generation of Operational Amplifiers

**DOI:** 10.3390/s24123990

**Published:** 2024-06-20

**Authors:** Zhenxin Zhao, Jun Liu, Lihong Zhang

**Affiliations:** 1 Innovation Center for Electronic Design Automation, Hangzhou Dianzi University, Hangzhou 310018, China; zxzhao@hdu.edu.cn; 2 Department of Electrical and Computer Engineering, Memorial University of Newfoundland, St. John’s, NL A1C 5S7, Canada; lzhang@mun.ca

**Keywords:** automated Op-Amp generation, deterministic sizing, differential evolution

## Abstract

Operational amplifiers (Op-Amps) are critical to sensor systems because they enable precise, reliable, and flexible signal processing. Current automated Op-Amp generation methods suffer from extremely low efficiency because the time-consuming SPICE-in-the-loop sizing is normally involved as its inner loop. In this paper, we propose an efficiently automated Op-Amp generation tool using a hybrid sizing method, which combines the merits together from a deterministic optimization algorithm and differential evolution algorithm. Thus, it can not only quickly find a decent local optimum, but also eventually converge to a global optimum. This feature is well fit to be serving as an acute filter in the circuit structure evaluation flow to efficiently eliminate any undesirable circuit structures in advance of detailed sizing. Our experimental results demonstrate its superiority over traditional sizing approaches and show its efficacy in highly boosting the efficiency of automated Op-Amp structure generation.

## 1. Introduction

Op-Amps are crucial for amplifying low-level sensor signals to a usable range, ensuring high input impedance to prevent the loading of sensitive sensors, and providing low output impedance for driving downstream circuitry. Additionally, Op-Amps enable signal conditioning operations such as filtering, linearization, and level shifting, improving the accuracy and stability of sensor outputs. Moreover, Op-Amps excel in differential signal processing, precision amplification, and noise reduction, contributing to enhanced signal integrity and robustness in sensor systems [1]. From temperature sensing and strain measurement to biomedical monitoring and industrial automation, the applications of Op-Amps in sensors are vast, underpinning the foundation of modern sensor technology.

As existing Op-Amp reuse and manual design styles become increasingly more difficult to meet the emerging application requirements, the efficient automated generation of Op-Amps is in great demand, which aims to automatically generate the Op-Amp structure and derive the device parameter values that satisfy the target design specifications. However, the existing research and development outcomes on the automated Op-Amp generation commonly suffer from low efficiency, leading to a barrier to their industrial applications in practice. Specifically, the evolution algorithm (EA)-based [2,3] or graph construction (GC)-based [4,5] Op-Amp generation methods can easily produce millions of Op-Amp structures for evaluation during the generation process due to their stochastic or brute-force explorative generation style, respectively. Assessing the feasibility of such a huge number of circuit structures would readily cause extremely low efficiency since the time-consuming circuit sizing operation has to be performed on each of the generated circuit structures.

In order to promote efficiency, a structure-independent setup of the equation-based behavioral model [6] and numerical performance model [7] have been proposed to replace the computation-intensive SPICE simulator to cut down the circuit evaluation time. Moreover, some heuristic methods [8,9] or machine learning methods [10,11] have also been proposed to shrink the sizing iterations. However, although these methods indeed accelerate the circuit sizing process, they can hardly deal with such an explosive number of circuit structures generated. An effective way to promote efficiency is to quickly eliminate some undesirable circuit structures before they move to the time-consuming simulation-in-the-loop detailed sizing stage. Recently, the symbolic analysis-based fast evaluation [12], which can quickly filter out around half of the undesirable structures, has exhibited its strong efficacy. However, since this method can only perform a rough assessment, there is still a lot of room to further reduce undesirable circuit structures in advance of detailed sizing, thus improving the generation efficiency.

In this paper, a deterministic parameter updating algorithm is proposed to enhance the efficiency and effectiveness of the deterministic sizing algorithm originally proposed in [13]. Then, a hybrid sizing method is proposed based on our deterministic parameter-updating algorithm, which can quickly figure out a decent local optimum while holding the capability of converging to a global optimum. Lastly, our hybrid sizing method is applied as a filter in the circuit structure evaluation flow to significantly accelerate the efficiency of automated Op-Amp structure generation.

The rest of this paper is organized as follows. Section 2 presents our deterministic-parameter-updating algorithm. Section 3 shows the acceleration scheme of automated Op-Amp generation with the aid of our newly proposed hybrid sizing algorithm. Section 4 reports our experimental results and Section 5 concludes this paper.

## 2. Deterministic Parameter-Updating Algorithm

Deterministic sizing was originally proposed in [13], and it is targeted at optimizing the device sizes in a circuit with fixed biases. However, during the Op-Amp generation process, the produced circuit structures are neither sized nor biased. From our experiments, the sizing effectiveness is poor if it is directly applied to evaluate the produced circuit structures. To make it fit for the application scenario of automated Op-Amp generation, we propose a novel deterministic parameter-updating algorithm.

In this section, we will first introduce the preliminary knowledge about deterministic sizing. Then, we will describe our improvements in terms of efficiency and effectiveness, which are composed of the classifying and updating of design parameters, processing of performance gradients, and bias-aware weight scheme. These improvements work together to form our deterministic parameter-updating algorithm.

### 2.1. Preliminary

The key to deterministic sizing is to determine the parameter correction (i.e., size change) at each sizing iteration, which is originally calculated by using a generalized boundary curve (GBC) algorithm developed in [14]. Firstly, the performance gradient ***g*** of current device sizes ***p*** is calculated by linearizing circuit performance *f*(***p***) at ***p***:

(1) $$g=(f\left(p+∆p\right)-f\left(p\right))/∆p$$

Then, ***g*** is used to calculate a parameter correction ***x*** via the GBC algorithm, which has the following objective function:

(2) $$minimize:\;{\left(\sum \exp\left(-\alpha \cdot \epsilon \left(x\right)\right)\right)}^{2}+\lambda \cdot {\left\|x\right\|}^{2}$$

Here factor *α* is a positive constant for the scaling purpose, variable *λ* controls the weight of the squared Euclidean norm of ***x***, and *ϵ*(***x***) is the linearized error:

(3) $$\epsilon \left(x\right)=f\left(p\right)-f_{spec}+g\cdot x$$

where $f_{\;spec}$ refers to the performance specification. Once the *x* is derived, the device sizes are updated as below:

(4) $$p_{new}=p+x$$

**Figure 1:**
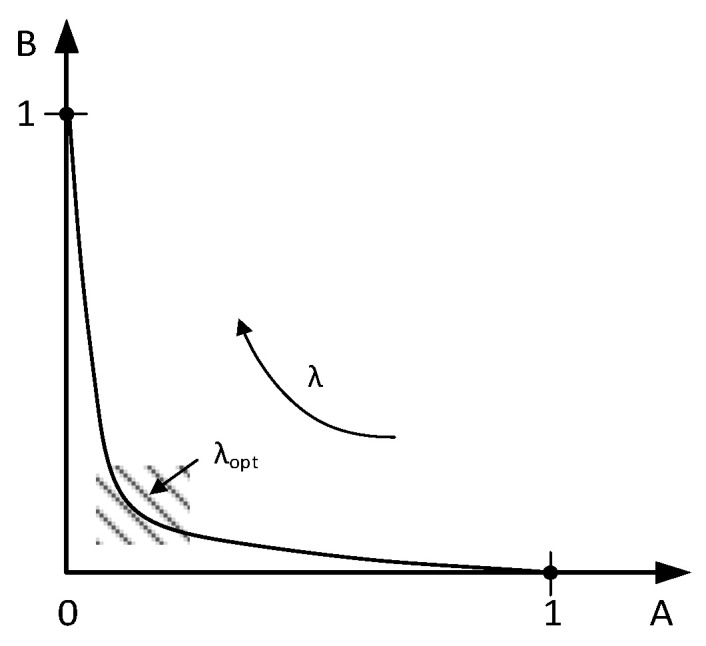
Boundary curve example.

To solve the minimization problem in (2), a typical boundary curve is plotted based on (2) and (3) by sweeping *λ*, as depicted in Figure 1 [14]. The *A* axis and *B* axis are transformed between 0 and 1 to represent the normalized parameter correction amount and the normalized objective improvement, respectively. The objective of a deterministic sizing algorithm is to maximize or minimize certain circuit performance attributes. When *λ* is infinite, according to (2), the parameter correction has to be 0 in order to minimize the cost function, and thus there is no improvement in the objective performance. When *λ* is 0, the parameter correction can be as large as possible. In such a situation, the objective may be greatly improved with a large linearized error, which is in conflict with minimizing the cost function. As claimed in [14], an optimal point is located somewhere in the shaded region of Figure 1, where the circuit sizes can be reasonably updated while effectively improving the objective with a significant error reduction.

The solution point can be found by first plotting the curve and then identifying the point with the smallest curvature radius. But intensive mathematical calculations and approximations, i.e., deriving a group of *λ* values for curve plotting after solving a nonlinear optimization problem, are required on the GBC extraction, which would lower the algorithmic efficiency. To efficiently derive it, a modified GBC algorithm, which employs the binary search method to efficiently determine the optimal *λ* and corresponding parameter correction, is proposed in [15]. However, in this algorithm, the Euclidean norm of performance gradient ***g*** (i.e., ‖***g***‖) rather than ***g*** is used to calculate the linearized error:

(5) $$\epsilon \left(x\right)=f\left(p\right)-f_{spec}+\|g\|\cdot x$$

It results in the solved parameter correction to be a scalar value (i.e., *x*). Based on it, the design parameters have to be updated in the way that each parameter in ***p*** will add this unified parameter correction value:

(6) $$p_{new}=p+x$$

### 2.2. Classifying and Updating of Design Parameters

The updating manner expressed in (6) induces a problem; that is, some design parameter values may be significantly changed after updating, but their performance gradients are actually small, leading to the nonlinear updating of these design parameters. To address this issue, a straightforward method is to calculate the parameter correction separately for each design parameter:

(7) $$\epsilon \left(x_{j}\right)=f\left(p_{j}\right)-f_{spec}+\|g_{j}\|\cdot x_{j}$$

where $x_{j}$ represents the parameter correction of the $j_{th}$ parameter in ***x*** and ‖$g_{j}$‖ refers to the performance gradient in terms of the $j_{th}$ parameter in ***p***. Then, the design parameter values are updated through (4). As can be seen, this method updates each design parameter with its own parameter correction at the cost of more computation required compared with the improved GBC method.

In this paper, we propose a new method to derive parameter updates. Firstly, we classify the design parameters according to their types, such as bias voltage, bias current, transistor length, transistor width, resistor, and capacitor. Then, the parameter correction is calculated for each type of design parameter with the following linearized error equation:

(8) $$\epsilon \left(x_{k}\right)=f\left(p_{k}\right)-f_{spec}+\|g_{k}\|\cdot x_{k}$$

Here $x_{k}$ represents the parameter correction of the $k_{th}$ type of design parameter and ‖$g_{k}$‖ refers to the performance gradient in terms of the $k_{th}$ type of design parameter. After that, each type of design parameter will add its corresponding parameter correction value (i.e., $x_{k}$):

(9) $$p_{k,new}=p_{k}+x_{k}$$

This method can be deemed as a trade-off between the two methods above, which try to balance efficiency and accuracy by updating design parameters based on their types, but this method actually features both better efficiency and accuracy than them.

### 2.3. Processing of Performance Gradients

After deriving the performance gradients of design parameters at each sizing iteration, one may observe a phenomenon: a few gradients may be significantly larger than the others due to the fact that circuit performance has distinct sensitivities with reference to parameter changes. These large gradients would dominate the value of ‖$g_{k}$‖, leading to the nonlinear updating of the other design parameters that have small gradients. To address this issue, the derived gradients have to be processed before they are used to calculate the parameter correction. The processing includes the following two steps: (1) divide the gradients into vectors based on their types of design parameters; and (2) for each gradient in each vector, we need to first calculate the mean value of the other gradients in this vector and then remove it from this vector if it is significantly (e.g., 10 times) larger than the mean value. Our proposed gradient processing operation ensures the smooth updating of design parameters, which can enhance the sizing efficacy.

### 2.4. Bias-Aware Weight Scheme

During the circuit generation process, the produced circuit structures are neither sized nor biased. Through our experimental studies, we have found that tuning the biases is more critical than adjusting device sizes when transistors work at improper regions, whose performance is usually far away from the specification. This observation has inspired us to bring forth the following bias-aware weight scheme:

(10) $$\left\{\begin{array}{c} w_{k\in B}=1,\;w_{k\in O}=0;\;\;\;\;\;\;\;\;\;\;if\;\left\{\begin{array}{c} f\left(p_{k}\right)\leq a*f_{Lspec}\\ f\left(p_{k}\right)\geq a*f_{Uspec} \end{array}or\right.\\ w_{k\in B}=1-({\frac{f\left(p_{k}\right)}{f_{spec}})}^{b},\;w_{k\in O}=({\frac{f\left(p_{k}\right)}{f_{spec}})}^{b};\;\;\;\;\;\;\;\;\;\;\;\;\;\;\;\;else \end{array}\right.$$

where $f_{Lspec}$ and $f_{Uspec}$ refer to the lower and upper bound performance requirements, respectively, *a* belongs to [0, 1), *b* is a natural number, and $w_{k\in B}$ and $w_{k\in O}$ refer to the weights of the $k_{th}$ type of design parameters that are bias-type (e.g., bias voltage, bias current, or device sizes of any bias circuits) and other-type design parameters, respectively. After adopting such a bias-aware weight scheme, the design parameter-updating Equation (9) has to be refined as follows:

(11) $$p_{k,new}=p_{k}+w_{k}\cdot x_{k}$$

As one can see, when the current performance is quite distant from the specification, only the bias-type design parameters can be tuned. Once it passes a certain threshold, both the bias-type and other-type design parameters are adjusted simultaneously with distinct weights. We will explore the best choices for the values of *a* and *b* in our experiments (Section 5).

Our proposed deterministic parameter-updating algorithm is illustrated in Algorithm 1, which integrates the three aspects as presented in Section 2.2, Section 2.3 and Section 2.4. Specifically, Line 3 calculates the gradients of design parameters while Line 4 classifies the design parameters based on their types. The for-loop depicted between Line 5 and Line 10 carries out our proposed performance gradient processing and bias-aware design parameter-updating method. The calculated parameter corrections are used to update design parameter values when the current performance distance is close to the previous one (Line 1). Otherwise, a smaller parameter correction value is directly derived and applied to update design parameter values (Line 12), which is newly defined in this paper.
**Algorithm 1**. Modified Deterministic Parameter-Updating Algorithm1.     **If** $\left|d_{t}-d_{t-1}\right|<\delta$: 2.           Lock current design parameter values with $p_{lock}=p_{t}$; 3.           Calculate the performance gradient $g_{t}$; 4.           Classify design parameters according to their types; 5.           **For** each type (index of k) of design parameters: 6.                  Process $g_{t}$ to get $g_{t,k}$ 7.                  Update the weight $w_{k}$; 8.                  Calculate the parameter correction $x_{k}$; 9.                  Update design parameters with $p_{t+1}=p_{t}+w_{k}\cdot x_{k}$; 10.          **End For** 11.    **Else**: 12.           $p_{t+1}=(1-\gamma )\cdot p_{t}+\gamma \cdot p_{lock}$; 13.    **End if**

## 3. Hybrid Sizing Algorithm Fit for Automated Op-Amp Generation

In this section, we will first present our proposed hybrid sizing algorithm and then illustrate its application to the circuit structure evaluation flow during the automated Op-Amp generation process.

### 3.1. Hybrid Sizing Algorithm

Starting from a given initial design point, the proposed deterministic parameter-updating algorithm is able to efficiently figure out the local optimal performance when one of the terminating conditions described in (12) and (13) is satisfied:

(12) $$f(p_{t+1})\left\{\begin{array}{c} \leq f_{Lspec},\;\;\;if{\;f}_{Lspec}\;is\;applied\\ \geq f_{Uspec},\;\;\;if{\;f}_{Uspec}\;is\;applied \end{array}\right.$$

(13) tolerence==θ

where *tolerance* is used to record the number of iterations that its performance does not get improved, and *θ* is an integer value. Our purpose of setting *tolerance* is to allow the deterministic sizing to escape from the local minimum to some extent.

However, since the exploration space of deterministic sizing is restricted by the initial design point, an inferior initial design point may lead to a poor sizing result in the end. To address this challenge, in this paper, we propose a novel hybrid sizing algorithm depicted in Algorithm 2. It borrows the grouping concept from the particle swarm optimization (PSO) algorithm, making the sizing algorithm start from a group of initial design points to exploit good local optimal results in a delving manner. On top of that, the mutation and crossover operations inspired by the differential evolution (DE) algorithm are integrated into the group-based deterministic sizing scheme to help it explore resources for the global optimum.
**Algorithm 2**. Hybrid Sizing Algorithm     **Input**: group size *G*; maximum number of iterations *N*; target performance specification               *Spec*; tolerance *θ*.     **Output**: circuit performance and its corresponding sizes. 1.   Randomly generates *G* sets of initial design points; 2.   Create *G* units of starting sizes with these initial design points; 3.   **While** (iteration index *t* < *N*): 4.         Evaluate the performance of each unit; 5.         Update *uBestScore* and set *state* of each unit; 6.         Update *gBestScore* and its corresponding solution of the group; 7.         **If** *gBestScore* meets *Spec*: **break**; 8.         Fill the group by using mutation and crossover operations; 9.         Parallelly update the design parameter values of each unit via Algorithm 1; 10.        *t* = *t* + 1; 11. **End While** 12. **Return** *gBestScore* and its corresponding solution;

As shown in Lines 1–2 in Algorithm 2, a group of units are created to start sizing with randomly generated initial design points. Each unit has three *states*: (1) finish; (2) stop; and (3) continue. The *state* is set based on Equation (14):

(14) $$\left\{\begin{array}{c} state=finish,\;\;\;\;\;\;\;\;\;\;\;\;\;\;if\;uBestScore\;meets\;Spec\\ state=stop,\;\;\;\;\;\;\;\;\;\;\;\;\;\;\;\;\;if\;tolerance==\theta \;\;\;\;\;\;\;\;\;\;\;\;\;\;\\ state=continue,\;\;\;\;\;\;\;\;\;else\;\;\;\;\;\;\;\;\;\;\;\;\;\;\;\;\;\;\;\;\;\;\;\;\;\;\;\;\;\;\;\;\;\;\;\;\;\;\;\;\;\;\;\; \end{array}\right.$$

where *uBestScore* refers to the best score that a unit has achieved so far, and *Spec* means the target performance specification. For each sizing iteration, all the units would be evaluated and set *state* first. Then, the best score of the group (i.e., *gBestScore*) and its corresponding design parameter values (i.e., sizes) are updated. After that, the *gBestScore* is compared with performance specification to decide whether the sizing process should be terminated or not (Line 7).

For the units whose states are *stop*, their associated deterministic sizing processes should be stopped. To maintain the same size of the group, each stopped unit would perform the mutation and binary crossover operations as described in (15) and (16) to produce a new unit, whose state would be reset as *continue* (Line 8).

(15) $$v_{i}=x_{i}+MR*\left(x_{best}-x_{i}\right)+MR*(x_{r1}-x_{r2})$$

(16) $$u_{i,j}=\left\{\begin{array}{c} v_{i,j},\;\;if\;rand\left(0,1\right)\leq CR\;or\;j=j_{rand}\\ x_{i,j},\;\;otherwise\;\;\;\;\;\;\;\;\;\;\;\;\;\;\;\;\;\;\;\;\;\;\;\;\;\;\;\;\;\;\;\;\;\;\;\;\;\;\;\; \end{array}\right.$$

In (15), ***x****_i_* is the sizes of the stopped unit, ***x***_*r*1_ and ***x***_*r*2_ are two distinct randomly selected units whose states are *continue*, ***x****_best_* refers to the sizes corresponding to *gBestScore*, ***v****_i_* is the sizes after mutation operation, and *MR* is a constant value referring to the mutation rate. In (16), *CR* refers to the crossover rate. ***x****_i_* and ***v****_i_* are performed with binary crossover operation to produce ***u****_i_*, which is set as the initial design point for the newly created unit. The final step at each iteration is to update the design parameters of all the units separately through parallel computing (Line 9).

As one can see, the design parameters of all the units in the group are updated in a deterministic manner, respectively, while the mutation and crossover operations help the group figure out the global optimum, which contributes to our hybrid sizing algorithm.

### 3.2. Hybrid Sizing-Based Evaluator for Automated Op-Amp Generation

The work [12] includes a circuit structure evaluation flow which is composed of a hash table, a filter, and two evaluators. Among them, the hash table guarantees that one circuit structure only needs to be evaluated once. Specifically, each encoded circuit structure to be evaluated will be put into a bucket according to its hash. Since there are various encoding ways in the existing circuit generation works, we will not list a hash function here. For the circuit structures that have been fast evaluated by using symbolic analysis approaches, only their performance results are stored in the hash table. For the evaluator that can also determine device sizes, both their performance results and device sizes are stored in the hash table. The first evaluator is based on the symbolic analysis of the small-signal model [16], in which the GPDD algorithm [17] is employed to numerically calculate the performance of unsized circuit structures. But due to approximate calculation, this evaluator can only roughly assess the DC gain. If the evaluated performance results meet a given requirement, the structure passes the first filter and is further assessed. The multi-objective optimization is the second evaluator, which is carried out by the well-known NSGA-II algorithm [18]. It uses SPICE simulation to evaluate circuit performance, which ensures accuracy but at the cost of more evaluation time.

**Figure 2:**
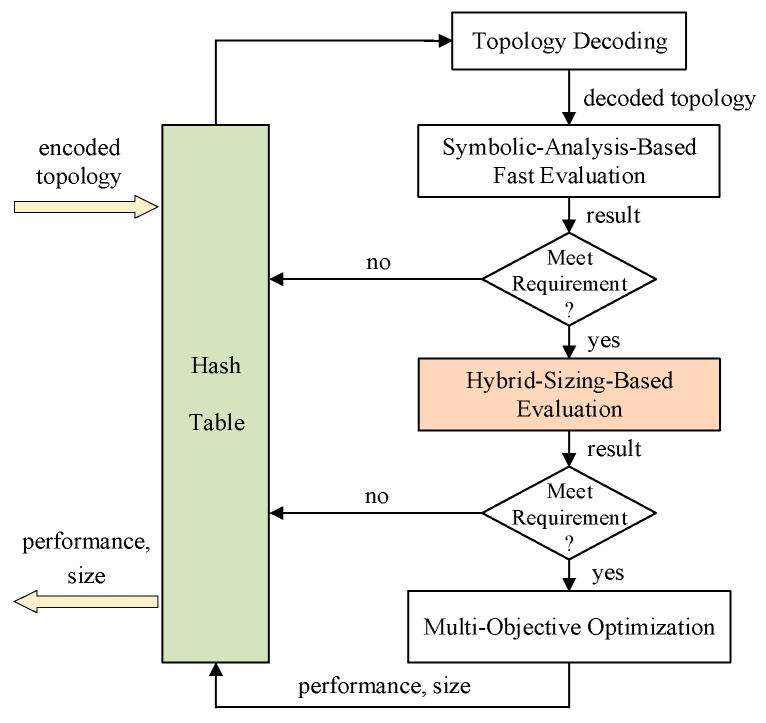
Our improved circuit structure evaluation flow.

In this paper, we add an evaluator and a filter into the circuit structure evaluation flow as shown in the shaded blocks of Figure 2. The evaluator is based on our proposed hybrid sizing method, which can quickly figure out a descent local optimal performance and its corresponding design parameter values. This local optimal can be utilized to filter any circuit structures whose performance does not meet a given requirement, while the optimized design parameter values can be treated as the initial design points for further evaluation, thus significantly boosting the efficiency of automated Op-Amp generation.

## 4. Experiments

The proposed hybrid sizing method and circuit structure evaluation flow were implemented in C++ with the SPICE simulations conducted by the Cadence tool (Cadence, San Jose, CA, USA). Our experiments were run on an Intel Xeon Silver 2.4-GHz Linux workstation that has 512 G of memory (Intel, Santa Clara, CA, USA). All the experiments were conducted in a CMOS 65-nm technology process.

### 4.1. Analysis of Deterministic Parameter-Updating Algorithm

**Figure 3:**
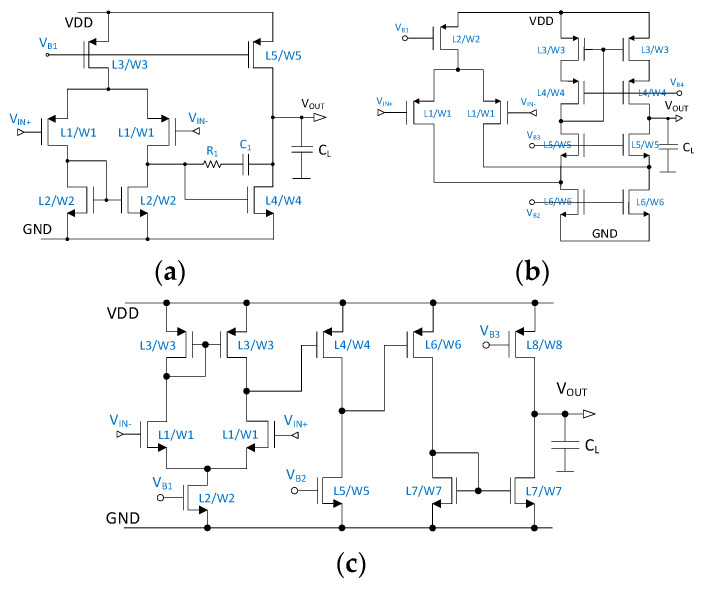
Test circuits: (**a**) two-stage Op-Amp; (**b**) folded-Cascode Op-Amp; (**c**) three-stage Op-Amp.

Our experiments were conducted on three test circuits: a two-stage Op-Amp, a folded-Cascode Op-Amp, and a three-stage Op-Amp, which have 12, 17, and 20 design parameters in total, respectively. These design parameters are composed of bias voltages, transistor sizes, capacitance, and resistance, which are labeled in blue in Figure 3. The efficacy of the deterministic sizing strongly relies on the initial design point. For fair analysis, we randomly generated 20 sets of design points for each test circuit, which were used as the initial design points for analyzing the proposed deterministic parameter-updating algorithm.

Firstly, we compare the three methods of calculating parameter correction and updating design parameters discussed in Section 2.2. By setting the target specification as 50 dB DC gain (*Av*) and the maximal number of allowed updating as 40, those 20 initial design points were applied to obtain the average performance of each method. Table 1 lists the experimental results, where *unified-x*, *individual-x*, and *our-x* refer to calculating parameter correction *x* with Equations (6), (4) and (9), respectively. As one can see, compared with *unified-x*, *individual-x* has more cases reaching the target specification but at the cost of more runtime on average. Before the gradient process (i.e., *NGP* standing for no-gradient process in Table 1), *our-x* uses the least average runtime but achieves comparable performance (i.e., the number of initial design points reaching the specification) of *individual-x*. After the gradient process (i.e., *GP* in Table 1), the performance of *our-x* is even better, which indicates the necessity of this operation.

In this part, we will explore the best choices for the values of *a* and *b*, which are the coefficients used to control the updating weights of the design parameters as listed in (10). Based on *our-x* method, we set the target specification as a quite large value that can never be reached and let all the sizing experiments run 40 iterations. Then, we fixed *a* as 0.5 and set *b* as 1, 2, 3, or 4 to check the maximal *Av* that these initial design points can achieve by applying our proposed bias-aware weight scheme. The mean and variance of the derived maximal gains are summarized in Table 2. In general, the sizing algorithm more stably (i.e., less variance) achieves better performance (i.e., larger mean value) when the bias-aware weight scheme is integrated. Among those four choices, *b* = 3 performs the best for all three test circuits. Due to this reason, *a* = 0.5 and *b* = 3 are set as the default values in the following experiments.

### 4.2. Analysis of Hybrid Sizing Algorithm

In this part, we will show the powerfulness of the proposed hybrid sizing algorithm through the comparison with the traditional PSO [19] and DE [20] sizing algorithms. Moreover, the group-based method (called *GroupDeter*), which has almost the same sizing steps as the hybrid sizing algorithm (called *Hybrid*) excluding mutation/crossover operations for producing new individuals (Line 8 in Algorithm 2), was also implemented for comparison.

In our experiments, we set the population size for DE and the group size for hybrid, PSO, and GroupDeter as 20, the maximal number of iterations as 40, the tolerance *θ* as 5, the mutation rate *MR* and crossover rate *CR* for hybrid and DE as 0.6 and 0.7, respectively, the inertia weight *w*, and acceleration coefficients *c*_1_ and *c*_2_ for PSO as 0.9, 2, and 2, respectively. The setting of these parameters is summarized in Table 3. A total of 20 sets of the design parameter values of the three test circuits were randomly generated and set as the initial design points for the individuals in all four algorithms. During the sizing process, the computation of individuals in a group or population was parallelly conducted for all these four algorithms to boost efficiency.

**Figure 4:**
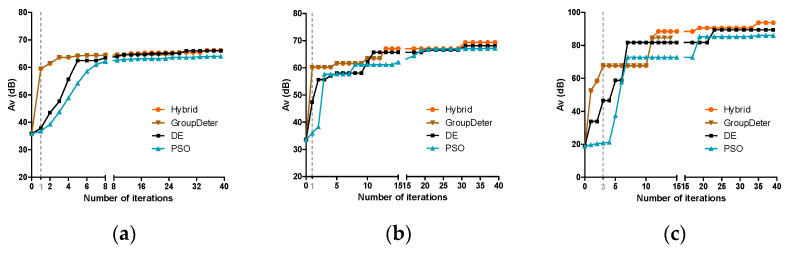
Sizing results of four sizing algorithms for (**a**) two-stage Op-Amp; (**b**) folded-Cascode Op-Amp; (**c**) three-stage Op-Amp.

We set the target performance specification as 100 dB *Av* and let the algorithm run 40 iterations. The sizing results of the three test circuits are depicted in Figure 4. As one can see, *Hybrid* and *GroupDeter* only use less than 3 iterations to figure out a quite good local optimum (e.g., around 60 dB *Av*) while *DE* and *PSO* need many more iterations to reach the same level of performance. Furthermore, *Hybrid* converges to a better global optimum than *DE* and *PSO* for all three test circuits. Without producing new units to fill the group, *GroupDeter* terminates when all its individual deterministic sizing processes are stopped, at the 27th, 13th, and 16th iteration for two-stage Op-Amp, folded-Cascode Op-Amp, and three-stage Op-Amp, respectively. Compared with *Hybrid*, *GroupDeter* can only reach 64.5 dB for the two-stage Op-Amp, 63 dB for the folded-Cascode Op-Amp, and 84.6 dB for the three-stage Op-Amp. These results demonstrate the advancement of our proposed hybrid sizing algorithm.

### 4.3. Analysis of Hybrid Sizing-Based Evaluator (HSE)

The reinforcement learning-based circuit synthesis framework presented in [12] was employed in our experiments to automatically generate Op-Amps. Six attributes are contained in our performance specification, which are quiescent power consumption (Power), DC gain (*Av*), unity-gain bandwidth (*UBW*), phase margin (*PM*), gain margin (*GM*), and slew rate (*SR*). The detailed performance specification is listed below:
  minimize*Power*
  maximize*Av*
  s.t.*Power* < 0.1 mW

*Av* > 60 dB

*UBW* > 10 MHz                  (17)
*PM* > 60°

*GM* > 10

*SR* > 15 V/µs


Since the above circuit synthesis algorithm [12] features a stochastic nature, we ran the algorithm 10 times as reported in Table 4 to average out random fluctuations. In Table 4, the symbol “*#*” refers to the number of occurrences while *Avg. Time* stands for the average runtime. As one can see, the hash table dramatically reduces the number of circuit structures to be evaluated from at least 300,000 to just more than 100, which means most of the evaluations were actually performed on the same structures during the Op-Amp generation process. The symbolic analysis-based evaluator is quite powerful, and it only consumed around 0.45 s on average but filtered more than half of the structures with the threshold setting as 30 dB *Av*. The threshold value for HSE was set as 55 dB *Av*. As observed from Table 3, the proposed HSE helps to promote generation efficiency in two ways: (1) eliminate more than half of the circuit structures to be further evaluated; and (2) reduce the runtime of multi-objective optimization (i.e., from around 28.5 min to about 22.5 min). If HSE is excluded in the evaluation flow, the produced circuit structures would be directly fed to the multi-objective optimization, leading to the average runtime of circuit generation being more than 28 h. However, after including HSE, the runtime decreases to around 9.25 h, which exhibits a significant boost of the generation efficiency by 3X times. Moreover, the experiments with such a big number of generated Op-Amp structures confirm the strong stability of our proposed HSE under different circuit structural conditions.

## 5. Conclusions

In this paper, we proposed a novel hybrid sizing algorithm which features fast access to a sound local optimum, making it perfectly suitable as a filter in the circuit structure evaluation flow to improve the efficiency of automated Op-Amp generation. Moreover, as experimental results demonstrated, our proposed hybrid sizing algorithm also has a better capability of finding the global optimum compared with traditional PSO and DE algorithms. Since it falls into the category of SPICE-simulation-in-the-loop during the circuit generation process, the accuracy of the optimized results is guaranteed.

## Figures and Tables

**Table 1:** Comparison among three different methods of calculating parameter correction and updating design parameters.

Updating Methods	Unified-x	Individual-x	Our-x (NGP)	Our-x (GP)
*Two-stage Op-Amp*				
# Reached	14	14	18	19
# Avg. Runtime	21.46 s	35.77 s	21.74 s	19.48 s
*Folded-Cascode Op-Amp*				
# Reached	14	14	19	19
# Avg. Runtime	49.74 s	84.96 s	41.49 s	40.13 s
*Three-stage Op-Amp*				
# Reached	9	12	14	15
# Avg. Runtime	60.25 s	76.89 s	61.27 s	56.64 s

**Table 2:** Experimental results of the proposed bias-aware weight scheme on three test circuits.

Statistical Indicators	Without Bias-Aware	a = 0.5 b = 0	a = 0.5 b = 1	a = 0.5 b = 2	a = 0.5 b = 3	a = 0.5 b = 4
*Two-stage Op-Amp*						
Mean (dB)	59.51	62.27	61.99	62.87	64.28	62.39
Variance	29.74	21.0	19.97	18.19	14.01	17.85
*Folded-Cascode Op-Amp*						
Mean (dB)	53.66	56.72	58.69	58.71	59.78	58.18
Variance	7.85	9.78	7.62	8.02	6.61	7.3
*Three-stage Op-Amp*						
Mean (dB)	57.43	62.02	61.97	63.72	67.59	63.54
Variance	19.86	15.73	16.2	13.02	11.08	13.69

**Table 3:** Parameters for the four compared algorithms.

Parameter	Value
*θ*	5
*MR*	0.6
*CR*	0.7
*w*	0.9
*c*_1_	2
*c*_2_	2

**Table 4:** Details of the circuit structure evaluation flow.

	Evaluations	In Hash Table	Symbolic Analysis	HSE	Multi-Obj. Opt.	Total
*Without HSE*						
#	325,675	325,524	171	-	59	-
Avg. Time	-	-	0.47 s	-	28 m 27 s	28 h 10 m
*With HSE*						
#	301,479	301,316	163	31	23	-
Avg. Time	-	-	0.41 s	26.73 s	22 m 36 s	9 h 15 m

## Data Availability

Data are contained within the article.
